# Mitochondrial Mutations Can Alter Neuromuscular Transmission in Congenital Myasthenic Syndrome and Mitochondrial Disease

**DOI:** 10.3390/ijms24108505

**Published:** 2023-05-09

**Authors:** Kaela O’Connor, Sally Spendiff, Hanns Lochmüller, Rita Horvath

**Affiliations:** 1 Children’s Hospital of Eastern Ontario Research Institute, Ottawa, ON K1H 8L1, Canada; 2 Department of Cellular and Molecular Medicine, University of Ottawa, Ottawa, ON K1H 8M5, Canada; 3 Centre for Neuromuscular Disease, University of Ottawa Brain and Mind Research Institute, Ottawa, ON K1H 8M5, Canada; 4 Division of Neurology, Department of Medicine, The Ottawa Hospital, Ottawa, ON K1H 8L6, Canada; 5 Brain and Mind Research Institute, University of Ottawa, Ottawa, ON K1H 8M5, Canada; 6 Department of Neuropediatrics and Muscle Disorders, Faculty of Medicine, Medical Center—University of Freiburg, 79104 Freiburg, Germany; 7 Centro Nacional de Análisis Genómico (CNAG-CRG), Center for Genomic Regulation, Barcelona Institute of Science and Technology (BIST), 08028 Barcelona, Catalonia, Spain; 8 Department of Clinical Neurosciences, University of Cambridge, Cambridge CB3 0FD, UK

**Keywords:** mitochondrial disease, congenital myasthenic syndrome, mitochondria, neuromuscular junction, neuromuscular, SLC25A1, TEFM

## Abstract

Congenital myasthenic syndromes (CMS) are a group of rare, neuromuscular disorders that usually present in childhood or infancy. While the phenotypic presentation of these disorders is diverse, the unifying feature is a pathomechanism that disrupts neuromuscular transmission. Recently, two mitochondrial genes—SLC25A1 and TEFM—have been reported in patients with suspected CMS, prompting a discussion about the role of mitochondria at the neuromuscular junction (NMJ). Mitochondrial disease and CMS can present with similar symptoms, and potentially one in four patients with mitochondrial myopathy exhibit NMJ defects. This review highlights research indicating the prominent roles of mitochondria at both the pre- and postsynapse, demonstrating the potential for mitochondrial involvement in neuromuscular transmission defects. We propose the establishment of a novel subcategorization for CMS—mitochondrial CMS, due to unifying clinical features and the potential for mitochondrial defects to impede transmission at the pre- and postsynapse. Finally, we highlight the potential of targeting the neuromuscular transmission in mitochondrial disease to improve patient outcomes.

## 1. Introduction

Congenital myasthenic syndromes (CMS) are a heterogenous group of rare, heritable disorders characterized by impaired transmission at the neuromuscular junction (NMJ). Classically, CMS presents during infancy or childhood with fatigable muscle weakness and are commonly associated with ptosis and ophthalmoparesis [1]. The phenotypic presentation, disease progression, and severity are all highly dependent on the genetic cause, and manifestations can vary widely. Many CMS subtypes are responsive to treatment, but drugs that may result in improvement for one genetic subtype of CMS can be detrimental for another type. Therefore, understanding the exact mechanism of the NMJ defect is of the utmost importance. Common treatment options include acetylcholinesterase inhibitors (AChE inhibitors), which increase the available acetylcholine (ACh) at the synapse, and 3,4-diaminopyridine (3,4-DAP), which increases the release of ACh by blocking potassium channels. The β-adrenergic agonists, salbutamol and ephedrine, are other therapeutic options for certain subtypes of CMS—these may act by slowly improving morphological defects at the postsynapse [2].

To date, 35 causative CMS genes have been identified; however, as whole exome/whole genome sequencing becomes more attainable, novel genes continue to be discovered [1]. Current identified genes are typically classified into four subcategories: synaptic, presynaptic, postsynaptic, and glycosylation-related. While most genes can be classified based on where their pathomechanisms occur, glycosylation is ubiquitous, and, thus, it remains unclear exactly where impairments arise. Similarly, two novel mitochondria-associated genes were recently identified as genetic causes for CMS: mitochondrial transcription elongation factor (TEFM) and the mitochondrial citrate carrier solute carrier family 25 member 1 (SLC25A1). It remains unclear how these mutations fit into the current classification categories. Understanding mitochondrial mutations with NMJ dysfunction provides insight into what commonalities may be present in this specific presentation of a CMS.

Despite evidence demonstrating the importance of mitochondria at the NMJ, the potential impact of mitochondrial protein mutations on neuromuscular transmission is frequently overlooked. Nevertheless, there is evidence of NMJ transmission defects caused by mutated mitochondria-associated genes in patients with mitochondrial diseases. Recently, in a cohort of various forms of genetically confirmed primary mitochondrial disease, electrophysiological studies detected NMJ abnormalities in over 25% of patients [3]. These cases present the opportunity to assess the roles of mitochondria that are critical for NMJ transmission, as well as highlight the potential for novel treatment avenues in mitochondrial patients by targeting the NMJ. Additionally, considering mitochondrial genes in patients clinically presenting with CMS may aid the diagnostic yield. Underscoring the link between mitochondria and CMS allows for innovations in one field to potentially be applied to the other. This may provide novel treatment options for mitochondrial disease and benefit patients with CMS.

This review will provide an overview of neuromuscular transmission and the role of mitochondria in this process. We focus primarily on the roles of mitochondria in energy production via the generation of ATP by oxidative phosphorylation and calcium (Ca^2+^) regulation. In our discussion of clinical presentations, we propose a novel subtype classification for CMS—mitochondrial CMS—using the recently identified causative CMS genes TEFM and SLC25A1. We also highlight the need to assess NMJ defects in traditional mitochondrial disease patients as a large proportion of patients may experience treatable fatigue. Understanding the role of mitochondria at the NMJ is critical for the proper care, diagnosis, and treatment of a subset of patients with NMJ dysfunction.

## 2. The Role of Mitochondria at the Neuromuscular Junction

**Figure 1:**
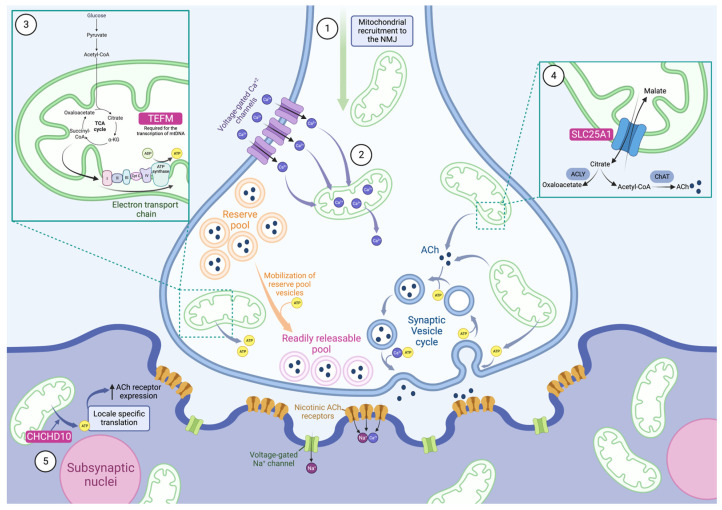
Mitochondria play a multi-faceted role at the neuromuscular junction. Evidence shows that mitochondria are actively recruited to the neuromuscular junction (NMJ), and they are present at much higher concentrations here than in nearby areas of the axon (1). As an action potential arrives at the presynapse, voltage-gated Ca^2+^ channels open and allow the rapid influx of Ca^2+^. Mitochondria buffer these high levels of Ca^2+^ by sequestering the ions and slowly releasing them (2). This helps control the release of synaptic vesicles as exocytosis is triggered by Ca^2+^. Simultaneously, mitochondria produce large amounts of ATP by oxidative phosphorylation to provide energy for the demanding processes triggered by action potentials (3). This increased ATP synthesis is modulated by Ca^2+^ levels. ATP is required for the mobilization of reserve pool vesicles as well as numerous stages of the synaptic vesicle cycle, including exocytosis and reacidification, with endocytosis requiring the bulk of it. TEFM, a causative CMS gene, is required for the transcription of mitochondrial DNA (mtDNA), including proteins involved in oxidative phosphorylation (3). Another causative CMS gene is SLC25A1, which is a mitochondrial citrate carrier and allows for the efflux of citrate from mitochondria (4). Citrate is cleaved into oxaloacetate and the ACh precursor, acetyl-CoA, by ATP citrate lyase (ACLY). We hypothesize that this is how SLC25A1 contributes to NMJ defects. After the Ca^2+^-triggered exocytosis of synaptic vesicles and the release of ACh into the synapse, postsynaptic nicotinic ACh receptors open, allowing the influx of cations into the motor endplate. This depolarizes the membrane, triggering voltage-gated Na^+^ channels, and produces a new action potential. CHCHD10 was shown to play a role in the postsynaptic functioning of the NMJ, presumably by providing ATP via oxidative phosphorylation to enable locale specific translation (5). This increases the ACh receptor expression and enables the transmission of the presynaptic signal. Mitochondria play complex roles at the NMJ at both the pre- and postsynapse. It is thus unsurprising that impaired mitochondria, including cases caused by mutations to SLC25A1, TEFM, and CHCHD10, can cause NMJ dysfunction.

NMJs are the synapses between motor neurons and skeletal muscle. Their basic function and organization have been reviewed extensively and are briefly summarized here (and reviewed in [4,5]) (Figure 1). Lower motor neurons originate in the spinal cord and form excitatory synapses with skeletal muscle mediated by acetylcholine (ACh) in humans and other mammals. As an action potential arrives at the presynaptic terminal, voltage-gated Ca^2+^ channels allow the influx of Ca^2+^ into the cytosol. This calcium triggers the exocytosis of synaptic vesicles to release ACh into the synaptic cleft. There, ACh interacts with the acetylcholine receptors (AChRs) at the motor endplate, triggering an influx of positive ions into the muscle. The membrane potential generated by this influx triggers the depolarization and opening of voltage-gated sodium (Na^+^) channels to produce a new impulse. The action potential travels through the muscle fiber until it reaches the sarcoplasmic reticulum, causing the release of calcium, which leads to muscle contraction. Mitochondria are actively recruited to the synapse and play important roles in its formation, energy production, and Ca^2+^ regulation.

The knowledge that mitochondria can have an impact on neuromuscular transmission, in both CMS and mitochondrial disorders, broadens our understanding of the varied roles that mitochondria play at the NMJ. This section will highlight some of the key features that have been linked to mitochondria and neuromuscular transmission.

### 2.1. Role of Mitochondria in the Formation of the NMJ

Mitochondria play an important role at the presynapse and are actively recruited to the area [6] (Figure 1). Mitochondria are highly enriched at NMJ presynapses, and nerve stimulation increases their localization to the axon terminals, which is critical for synaptic potentiation and differentiation [6,7,8,9,10]. The enrichment of mitochondria at the NMJ presynapse is one way in which the cells adapt to increased energy demand, and, in parallel, there is an increase in the number of synaptic vesicles [8]. The high number of mitochondria at the synapse with elevated membrane potentials indicates an increased capacity for ATP production [8,10]. Comparing the NMJ presynapse region to the surrounding axon reveals 72% more mitochondria at the motor presynapse, which is not observed in sensory neurons [9]. This indicates that there is a unique relationship between the NMJ and mitochondria that is not necessarily observed at all synapses.

Localization to the distal end of axons begins during the outgrowth period, when mitochondria are not only enriched at the growth cone but are also specifically delivered there [11,12]. Without mitochondria at the growth cone, outgrowth is significantly impaired [11,12,13]. The overexpression of mitochondria at the growth cone improves the process [14]. Specifically, ATP production at the growth cone is most likely used in actin dynamics, and mitochondria are expected to contribute to this process [14,15,16]. There is a demand for oxidative phosphorylation for neurite outgrowth, and a local increase in mitochondrial biogenesis is required [14]. Finally, mitochondria also present a risk to axon outgrowth due to their production of reactive oxygen species (ROS). Impaired mitochondria can result in the increased production of ROS and lead to a reduction in axon outgrowth [13,17]. The role of mitochondria in axon outgrowth is particularly relevant as both mitochondrial CMS zebrafish models, TEFM and SLC25A1, have been shown to display abnormal axon outgrowth [18,19].

### 2.2. Mitochondrial Ca^2+^ Regulation at the Presynapse

With the arrival of the action potential at the presynapse, large amounts of Ca^2+^ enter the cell through voltage-gated Ca^2+^ channels. The cytosolic Ca^2+^ concentration rises rapidly, before slowing and reaching a plateau. Cytosolic Ca^2+^ concentration is buffered by the rapid uptake of Ca^2+^ into the mitochondria before the concentration plateaus in both subcellular locales [20,21,22] (Figure 1). Mitochondria simultaneously intake Ca^2+^ and slowly release it during stimulation; Ca^2+^ release then occurs more rapidly after the stimulation is over [23,24,25].

Importantly, normal NMJ function relies on the sequestration of Ca^2+^ in mitochondria. Ca^2+^ is the molecular cue for exocytosis, and, therefore, its levels influence the release of synaptic vesicles. The influx of Ca^2+^ to the mitochondria is not dependent on ATP production, but it does rely on membrane polarization [26]. The membrane depolarization of mitochondria results in an impaired Ca^2+^ influx, and cytosolic Ca^2+^ concentrations rise to amplitudes far higher than they do under standard physiological conditions [25,27,28,29]. This shows how important mitochondrial uptake is to balance and sustain stimulation without Ca^2+^ overload.

In a mouse model of spinal muscular atrophy (SMA), impaired transmission and synaptic vesicle release were associated with fewer mitochondria and a diminished ability to sequester Ca^2+^ compared to controls [30,31,32]. The impaired mitochondrial Ca^2+^ intake in these mice caused the abnormal release of ACh, impaired synaptic vesicle cycling, and depleted the quantal content more rapidly [26,30,33]. Synaptic vesicle cycling at the NMJ has been shown to primarily rely on oxidative phosphorylation, which is modulated by Ca^2+^ levels—therefore, Ca^2+^ likely plays a role in this process [9,30,34]. Interestingly, central nervous system (CNS) synapses rely on both glycolysis and oxidative phosphorylation [9,35]. Mitochondria are present in every NMJ presynapse but only in 50% of CNS synapses, which may explain why NMJs are particularly sensitive to mitochondrial dysfunction [9,10,36,37]. These results indicate that mitochondrial Ca^2+^ buffering is required for normal neuromuscular transmission. This reliance is especially evident during periods of high frequency stimulation and may explain the common exercise intolerance and fatigue in patients with mitochondrial dysfunction [22,23,25,38].

While less described, the oversaturation of mitochondria with Ca^2+^ can also damage NMJ function. This has been demonstrated in SOD1 mutant mice who have abnormal mitochondria with various alterations to Ca^2+^ influx activities, including increased Ca^2+^ concentration plateaus [22]. Evidence from CNS neurons indicates that Ca^2+^ overload in mitochondria can result in the production of ROS, which in turn cause damage to the oxidative phosphorylation system [39]. Furthermore, while an increased Ca^2+^ concentration within a normal physiological range stimulates the production of ATP, moderate to high Ca^2+^ overload inhibits ATP synthesis [40,41].

While all cells rely on mitochondria, their localization to synapses, including NMJs, is particularly important as energy and Ca^2+^ needs must be addressed locally [42]. Mitochondria are transported along microtubules towards the synaptic terminal via anterograde movement mediated by kinesin and towards the cell body via retrograde movement mediated by dynein. Miro is a mitochondrial outer-membrane protein, which is bound to the kinesin heavy chain by Milton. This interaction has been demonstrated in Drosophila and mammals, including humans [42,43,44,45]. This transport was shown to rely on Ca^2+^ in cortical rat neurons, where high Ca^2+^ concentrations impair the motility of mitochondria [42]. This is thought to be a mechanism for positioning mitochondria where they are most needed as increased Ca^2+^ cytosolic concentration is likely to need Ca^2+^ buffering.

Drosophila models demonstrated that mutant Miro (human homologs: RHOT1/2) disrupts the normal localization patterns of mitochondria in neurons and muscle by affecting both anterograde and retrograde movement [44]. In neurons, the mitochondria remain in neuronal cell bodies, and larval NMJs have no presynaptic mitochondria. These mutants show abnormal muscle size and NMJ morphology in addition to impaired neurotransmitter release and Ca^2+^ buffering under high-frequency stimulations. The neuronal, but not muscular, expression of wild-type Miro rescued some of these defects, including the fatigued release of neurotransmitters. These results demonstrate the importance of mitochondria at the presynapse in neurons. Notably, Drosophila are good models for presynaptic NMJ defects; however, because they use glutamate as a neurotransmitter rather than ACh, their relevance for postsynaptic modeling is limited [7].

### 2.3. Mitochondrial ATP Production at the Presynapse

ATP is the primary cellular energy source and is mostly produced by oxidative phosphorylation in the mitochondria. Glycolysis can also contribute to ATP production; however, it is much less efficient [30]. While glycolysis produces two ATP molecules per glucose molecule, oxidative phosphorylation can generate thirty. This highly efficient process is made possible by a series of redox reactions [46]. The citric acid cycle in the mitochondrial matrix generates electron donors, NADH and FADH2, which, when oxidized, donate electrons to the respiratory chain. A proton gradient is established across the inner mitochondrial membrane, and this energy is harnessed by ATP synthase to generate ATP (Figure 1). Upon neuronal stimulation, the mitochondrial capacity for ATP production is increased by increased mitochondrial matrix pH, inner membrane potential, and mitochondrial NADPH levels. This stimulation-induced mitochondrial energy production is mediated specifically by Ca^2+^, as shown in experiments that replaced Ca^2+^ with Sr^2+^, which demonstrated a sharp decline in the enhanced oxidative phosphorylation [47]. The role of Ca^2+^ in regulating ATP synthesis lies partially in its ability to induce the citric acid cycle (reviewed in [48]).

Action potentials trigger many energetically demanding processes and, therefore, require a large source of ATP. The Ca^2+^ influx that occurs upon action potential arrival rapidly increases oxidative phosphorylation, resulting in increased ATP production [34,47,49,50]. In fact, the novel generation of ATP is required in hippocampal neurons as, despite high resting levels of ATP, this is not sufficient for stimulation-induced function [35]. Ca^2+^ triggers the exocytosis of synaptic vesicles clustered in readily releasable pools (RRP) that are primed for release by ATP [35] (Figure 1). The fusion of the vesicles to the presynaptic membrane may also require ATP [51]. As exocytosis is established, endocytosis followed by the reacidification of the vesicles immediately begins—replenishing the RRP throughout tetanic stimulation [52]. During intense stimulation, synaptic vesicles from the reserve pool (RP) are mobilized for release upon the depletion of the RRP [53]. The RP is replenished only after stimulation has ended [52].

Evidence from disease models highlights the importance of mitochondrially derived ATP in RP mobilization. In a PINK1 Parkinson’s disease Drosophila model, rapid stimulation caused impaired NMJ synaptic transmission because of damaged RP mobilization [54]. Parkinson’s disease patients have been shown to have a decremental signal on repetitive nerve stimulation (RNS); however, they do not display altered jitter by single-fiber EMG (SFEMG) [55]. One potential mechanism for this impaired release from the RP is the ATP-dependent F-actin cytoskeleton that translocates vesicles from the RP to the RRP [8]. Disruption to this system has been shown to impact the RP but not the RRP [52]. Mitochondria have also been shown to contribute to releasing and replenishing the RRP; however, whether that mechanism relies on its ability to regulate calcium or to produce ATP, or a combination of the two, is not yet understood [56].

#### The Role of Mitochondria in Synaptic Vesicle Formation and Recycling

The synaptic vesicle cycle, composed of exocytosis, endocytosis, and reacidification steps, requires a large amount of energy (Figure 1). Endocytosis is particularly demanding, and ATP is needed to replenish the synaptic vesicle pools [37,57]. Reacidification and exocytosis seem to require less energy; however, they still contribute to ATP demand at the synapse and are required for normal function [37,50,51,58]. At the NMJ, oxidative phosphorylation is required for synaptic vesicle cycling, highlighting the role of mitochondria in normal neuromuscular transmission [30]. The inhibition of oxidative phosphorylation directly impairs synaptic vesicle cycling [50]. Leigh disease is a severe mitochondrial disorder, and one of the several genetic causes is mutations in the nuclear NDUFS4 gene, which encodes a subunit of the mitochondrial complex I. Knocking out this protein in mice resulted in lower levels of ATP and impaired synaptic vesicle cycling in hippocampal neurons [37]. Endocytosis was particularly sensitive to the loss of mitochondrially derived ATP. Leigh disease patients experience muscular symptoms including weakness and fatigue. To our knowledge, assessments of NMJ transmission in these patients has not been recorded in the literature. However, it may be interesting to study how mitochondrial defects impact the presynapse in patients with NDUFS4 mutations and in other genetic forms of Leigh Syndrome.

### 2.4. Mitochondrial Fusion-Fission at the NMJ

Mitochondria are dynamic organelles; they require fusion and fission to preserve their normal function. These processes are controlled by several proteins, including dynamin-1-like (DNM1L) protein, Mitofusin-2 (MFN2), Mitofusin-1 (MFN1), and optic atrophy 1 (OPA1) [59,60]. DNM1L is required for mitochondrial fission, while OPA, MFN1, and MFN2 are required for mitochondrial fusion.

In a Drosophila model, mutations in the DNM1L homolog, drp1, impeded the subcellular localization of mitochondria to the presynapse [53]. Despite an absence of mitochondria at the presynapse, transmission was normal during basal stimulation. However, upon high intensity stimulation, neuromuscular transmission was impaired [53]. The mechanisms behind the impairments seemed to vary based on the mutation within the gene: some drp1 mutations impair the recruitment and mobilization of vesicles but not synaptic vesicle cycling, while others impair cycling [53,61]. The mobilization of the RP was partially rescued by treating with exogenous ATP, indicating that the mechanism was reliant on mitochondrial ATP production rather than modulated by Ca^2+^ [53]. De novo heterozygous or biallelic variants in DNM1L have been associated with a severe mitochondrial disease causing delayed psychomotor development and hypotonia, which may lead to death in childhood. Many patients develop refractory seizures, consistent with an epileptic encephalopathy leading to neurological decline [62,63]. We were not able to find documentation of patients with DNM1L mutations being tested for neuromuscular transmission defects. We propose that there may be value in studying neuromuscular transmission in these patients in the future.

Mutations to MFN2 cause Charcot-Marie Tooth Disease Type 2A (CMT 2A), a disease characterized by variable but typically degenerative symptoms [64,65]. Animal models of other types of CMT have identified NMJ defects [66,67]. The loss of MFN2 from human embryonic stem cells caused mitochondrial fragmentation and defective mitochondrial functioning [68]. In addition to its role in mitochondrial fusion, MFN2 is also linked to mitochondrial transport via its interactions with Milton/Miro [69]. Impacts to the transportation of mitochondria were not directly related to fusion defects as the knockdown of OPA1 did not impact the process. Numerous studies indicate that mutations to or the loss of MFN2 causes axonal neuropathy via transportation deficiencies, rather than via its role in mitochondrial morphology dynamics [68,70,71].

Mutations to OPA1 most frequently causes optic neuropathy; however, patients often present with multi-systemic involvement including myopathy and peripheral neuropathy [72]. OPA1 patients have been investigated for impaired neuromuscular transmission, and two of seven patients displayed abnormal jitter [3]. The mechanism behind the involvement of the NMJ is not currently clear, although experiments using primary rodent cortical neurons demonstrated that low levels of OPA1 impact synaptic formation and maturation [73].

### 2.5. Mitochondria at the Postsynapse

Mitochondrial Ca^2+^ buffering and ATP synthesis work in conjunction to enable the normal functioning of the NMJ. Compared to the presynapse, there is less literature about the role of mitochondria at the motor endplate. However, it has been shown that, as in the presynapse, mitochondria are actively recruited to the postsynaptic terminal, specifically around the subsynaptic nuclei [74,75]. One possible explanation is that these mitochondria are required to provide energy for locale specific translation at the motor endplate. Most of the available information has focused on the mitochondrial protein CHCHD10, a protein implicated in disease and postsynaptic function at the NMJ.

#### CHCHD10 Plays a Role at the Postsynapse

CHCHD10 has been associated with a wide range of diseases including amyotrophic lateral sclerosis (ALS), frontotemporal dementia (FTD), Charcot-Marie Tooth disease, cerebellar ataxia, and mitochondrial myopathy [76]. This nuclear-encoded mitochondrial protein localizes to the intermembrane space, particularly at cristae junctions [77]. Importantly, it has been associated with respiratory chain dysfunction in patients [77].

One study indicated that CHCHD10 was primarily localized to the postsynapse and generated a skeletal muscle knockout mouse model of CHCHD10 [78]. These conditional knockout mice displayed neuromuscular transmission defects and morphological changes to the NMJ, including smaller clusters of AChRs. CHCHD10 was shown to be critical for mitochondrial ATP production in primary myotubes, and there is evidence that CHCHD10 contributes to AChR clustering by generating the ATP required for the enhanced expression of AChRs (Figure 1). As different CHCHD10 mutations cause motor neuron disease or frontotemporal dementia, CHCHD10 is likely important at the presynapse as well. Zebrafish CHCHD10 knockdowns display shortened axons, like in SLC25A1 and TEFM zebrafish models, demonstrating that CHCHD10 may play a role at both the pre- and postsynapse [79]. This highlights how the ubiquity of mitochondria may result in complex pathomechanisms occurring in multiple locations.

Other animal models of CHCHD10 demonstrate further evidence of motor deficits, reduced survival, and NMJ abnormalities [79,80,81]. In line with the association of CHCHD10 with neurodegenerative disease such as ALS, a mouse model expressing a patient mutation in CHCHD10 displayed progressive NMJ degeneration, including motor neuron loss [80]. Animal models have also demonstrated the deficient activity, assembly, and expression of numerous respiratory chain complexes [80,81]. These results provide a partial explanation as to why ATP production at the NMJ is impacted by CHCHD10 mutations; however, the mechanism is not yet fully elucidated.

While CHCHD10-related disorders in humans have not yet been documented to have neuromuscular transmission deficiencies, there is evidence that CHCHD10 plays a role at the NMJ. Therefore, it may be beneficial to study neuromuscular transmission in patients with CHCHD10 mutations, especially those diagnosed with mitochondrial myopathy or motor neuropathy. The identification of an NMJ defect in patients may provide novel therapeutic options, such as salbutamol or other drugs targeting the NMJ [19].

### 2.6. Summary of the Mitochondrial Role at the NMJ

Past work documents that the mitochondria play a role in neuromuscular transmission from development to function in both the pre- and postsynapse. Each of the roles described above can impact messaging from the nerve to the muscle, and the wide variety of involved processes and proteins indicates that the NMJ is vulnerable to mutations in any number of mitochondria-associated genes. Whether that be in Ca^2+^ signaling, the synthesis of ATP, proper NMJ development, or translation at the motor endplate, there are numerous opportunities for the involvement of mitochondria. Systematic studies of neuromuscular transmission in mitochondrial diseases may reveal further patients with a potentially druggable NMJ defect.

## 3. Impaired Mitochondrial Activity Can Impact Neuromuscular Transmission in Patients

**Figure 2:**
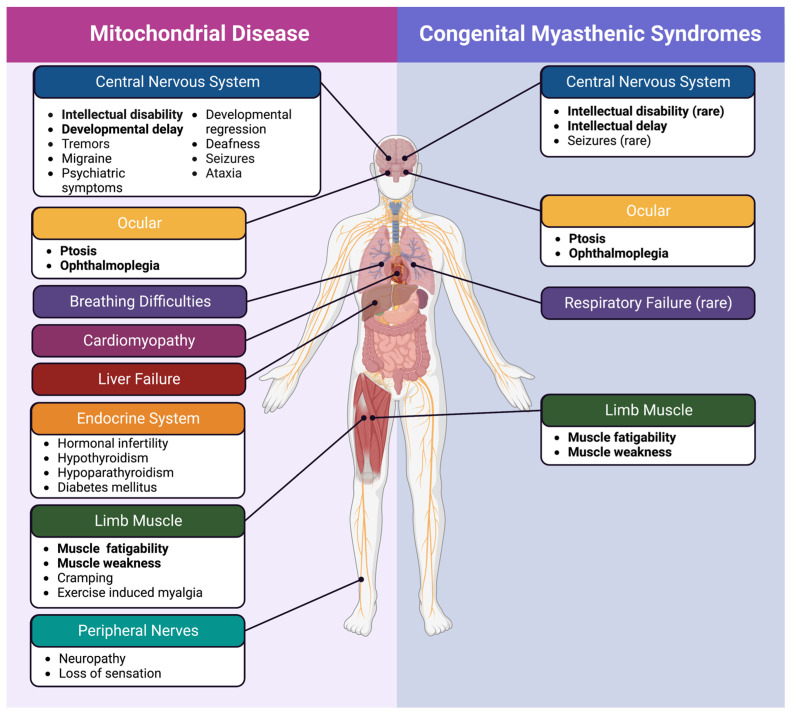
Mitochondrial disease and congenital myasthenic syndrome symptoms. Despite the involvement of mitochondria in both mitochondrial disease and mitochondrial CMS, there are still identifiable distinctions between the two. Symptoms that are observed in both disorders are bolded.

The presentation of CMS is highly heterogenous with a unifying feature of childhood-onset fatigable muscle weakness. The differential diagnoses include myasthenia gravis and mitochondrial myopathies [1] (Figure 2). An important diagnostic test in myasthenia gravis is the detection of antibodies in the serum against the AChR or other components of the NMJ (such as MusK). CMS is considered in patients who present with typical symptoms but are seronegative for myasthenia gravis autoantibodies and non-responsive to immunosuppressants [1,82,83]. Impaired neuromuscular transmission can be assessed by SFEMG, which detects abnormal neuromuscular jitter, and RNS, which detects decreased endplate potentials.

While a diagnosis of myasthenia gravis can frequently be excluded, the line between some subtypes of CMS and mitochondrial myopathy is less obvious. Recently, two nuclear encoded mitochondria-associated genes were identified as causative for CMS—SLC25A1 [18,84,85,86,87] and TEFM [19]. Notably, it is only specific “milder” genetic mutations to SLC25A1 and TEFM that cause CMS; other previously identified mutations are associated with more severe and classical mitochondrial disease. It has been recently shown that about one in four patients with mitochondrial myopathy have a significant defect of neuromuscular transmission [3]. These presentations demonstrate that more mitochondrial myopathy patients may have an NMJ defect, which could be targeted to improve fatigability or even weakness. On the other hand, patients with CMS, due to classic genetic causes such as mutations in DOK7 or COLQ, have been shown to display mitochondrial abnormalities [88,89], confirming the link between the NMJ and mitochondrial function. If new therapies in mitochondrial diseases emerge, these may also be considered in these forms of CMS.

Mitochondrial disease is caused by mutations in the mitochondrial DNA (mtDNA) or in ~400 nuclear genes impacting mitochondrial function. The clinical presentation, severity, age of onset, and prognosis of mitochondrial disease are all diverse, making it difficult to narrow down unifying features other than the underlying involvement of dysfunctional mitochondria (as reviewed in [90,91]). Patients may present with numerous symptoms such as deafness, myopathy, encephalopathy, ophthalmoplegia, stroke-like episodes, and more. The presence of multi-system involvement is typical for mitochondrial diseases as mitochondria are present in every human tissue; however, some forms of mitochondrial disease show very tissue-specific clinical presentations, and the reasons behind this are unknown. Symptoms such as fatigable muscle weakness, myalgia, and exercise intolerance are quite common among patients with mitochondrial myopathy and among patients with CMS, highlighting the phenotypic overlap between these conditions [90,91].

The overlap in clinical presentation, physiology, and, potentially, genetic causes between CMS and mitochondrial disease draws attention to the need for a better understanding of how mitochondria play a role at the NMJ in patients (Figure 2). To understand the role of mitochondria in the NMJ function, we explore current knowledge on CMS caused by mitochondria-associated genes.

### 3.1. Mitochondrial Congenital Myasthenic Syndrome

#### 3.1.1. SLC25A1 Mutations Can Cause CMS

SLC25A1 is a mitochondrial citrate carrier. It localizes to the mitochondrial membrane and allows for the efflux of citrate and the influx of cytosolic malate [92]. Importantly, citrate is cleaved into acetyl-coenzyme A (acetyl-CoA) and oxaloacetate by ATP citrate lyase (ACLY) (Figure 1). Acetyl-CoA, plays a significant role in the biosynthesis of fatty acids, cholesterol, bile salts, and hormones [92]. SLC25A1-related disease is typically associated with D2 and L2 hydroxyglutaric aciduria (D/L-2-HGA), a severe neurometabolic mitochondrial disease characterized by encephalopathy, severe muscle weakness, seizures, respiratory distress, psychomotor delay, and early death [93]. However, a subset of patients with SLC25A1 mutations have been identified with a less severe clinical presentation more representative of CMS, including fatigable muscle weakness and increased neuromuscular jitter [18,84,85,86,87]. As is commonly observed in CMS, patients had a variable presentation in addition to the fatigable weakness, including intellectual disability, epilepsy, and developmental delay. All but two CMS patients presented without the typical urine organic acids profile that is a signature of D/L-2-HGA [18,87]. Due to the severity and lethality of most D/L-2-HGA cases, it has been hypothesized that NMJ transmission dysfunction may occur in these patients as well but remain undetected, whereas patients with milder forms of the disease have more obvious NMJ dysfunction [18]. The heterogenous presentation of SLC25A1 mutations may be linked to the severity of the impact to the carrier function of the protein [18].

In one study, zebrafish SLC25A1-knockdown models demonstrated decreased spontaneous and touch-evoked escape responses [18]. NMJ staining revealed normal muscle morphology but short and erratic motor neuron end terminals. There was no evidence of normal synaptic formation, suggesting that SLC25A1 may act via a presynaptic mechanism.

The mechanism behind this mutant SLC25A1 driven neuromuscular transmission impairment has not yet been elucidated. It is known that acetyl-CoA is a necessary precursor of ACh. This process is catalyzed by choline acetyltransferase (ChAT), which is itself a causative CMS gene. We hypothesize that this may indicate that ACh production may be affected in SLC25A1 mutations (Figure 1). Acetyl-CoA can be derived from sources other than citrate, including glucose, beta-hydroxybutyrate, and acetate (as reviewed in [94]). However, the inhibition of ACLY has been shown to inhibit the production of ACh [95], ACLY localizes preferentially to cholinergic neurons [96,97,98], and it co-localizes with ChAT-expressing neurons in mice [99]. Further supporting this hypothesis is the evidence that some patients with SLC25A1 mutations are responsive to therapies that increase the accessibility of ACh, either via AChE inhibitors or 3,4-DAP [18,85,86,87]. SLC25A1 CMS patients display normal or only slightly impacted respiratory chain enzyme activity and normal ATP levels in muscle samples, but it remains undetermined whether this also reflects the situation at the presynapse [18,85].

#### 3.1.2. TEFM Mutations Can Cause CMS

TEFM is a mitochondrial transcription elongation factor that works in conjunction with the mitochondrial RNA polymerase (POLRMT) and two mitochondrial transcription initiation factors to transcribe mtDNA [100]. Essential oxidative phosphorylation subunits are encoded by mtDNA. TEFM is required to produce the long, polycistronic transcripts generated by normal mtDNA transcription [101]. A recent article described seven patients from five families presenting with TEFM mutations and neuromuscular disease, with extremely variable presentations including fatal neonatal presentation, severe epileptic encephalomyopathy, intellectual disability, and mitochondrial myopathy with NMJ dysfunction [19]. Two of these patients had been clinically diagnosed with CMS due to fatigable muscle weakness and transmission defects identified by RNS (c.469C>G; p.(Pro157Ala)). The other patients had not been tested for NMJ transmission defects. The variability in presentation may indicate that symptoms and severity are mutation specific as only the CMS sibling pair and one other pair had the same mutations (five unique genotypes). The two patients with a CMS phenotype were responsive to salbutamol, which is frequently used to treat NMJ transmission defects in CMS [2]. Additionally, the patients displayed evidence of mitochondrial myopathy, suggesting an overlap between neuromuscular transmission defects and mitochondrial disease. Skeletal muscle displayed abnormal mitochondria, reduced expression levels of oxidative phosphorylation subunits, and reduced the activity of oxidative phosphorylation complexes, reflecting a defect of mitochondrial protein synthesis [19] (Figure 1). TEFM mutations caused a decrease in mitochondrial translation elongation, leading to a severe defect of promoter-distal mt-mRNA, resulting in the reduced production of mtDNA-encoded proteins.

The causative impact of TEFM mutations was further demonstrated by the establishment of morpholino-induced knockdown and CRISPR-Cas9-induced knockout of tefm in zebrafish [19]. The tefm downregulated fish displayed movement defects and showed abnormal mitochondrial transcripts. The tefm knockdown zebrafish also demonstrated abnormal NMJs, with smaller clusters of synaptic vesicles and less co-localization between AChRs and synaptic vesicles. As seen in the zebrafish modeling of SLC25A1 disorders, there was aberrant neuron outgrowth. This is indicative of a presynaptic mechanism. Due to the reduced expression and activity of oxidative phosphorylation components, it seems likely that defects are caused by impaired mitochondrial transcription and the altered processivity of POLRMT resulting in reduced ATP production. As ATP is critical for neuronal outgrowth, NMJ formation, and synaptic vesicle clustering, this would also explain some of the morphological effects seen in the zebrafish [11,12,13]. The authors hypothesize that the variety in clinical presentation is likely dependent on the remaining activity in the mutated protein. The least clinically severe patients within this cohort were those diagnosed with CMS, indicating that the NMJ defect is part of the clinical presentation for this mitochondrial disorder.

#### 3.1.3. The Pathomechanism of Slow-Channel CMS May Involve Mitochondria

Slow-channel CMS is a subset of CMS caused by mutations to AChR subunits that result in the delayed closing of the channel causing cation overload at the postsynapse [1]. Motor endplate myopathy is frequently observed in addition to degenerating mitochondria and subsynaptic nuclei [102]. Interestingly, certain activated caspases, a family of proteases involved in apoptosis, are also localized more frequently to the postsynapse in this disorder [102,103]. Evidence suggests that these caspases are activated by degenerating mitochondria releasing cytochrome c after calcium overload [102,103]. Despite caspase levels not being correlated with disease severity in patients, inhibiting caspases relieves ultrastructural defects in mice [103]. While mitochondria are not the primary cause of this disorder, the mechanism does demonstrate that mitochondria can, and do, contribute to the pathology of other CMS subtypes.

### 3.2. Mitochondrial Disease Cases Display Signs of Transmission Defects

Mitochondrial disease, myasthenia gravis, and CMS have highly similar clinical presentations. While myasthenia gravis and CMS are both characterized by NMJ dysfunction, there is little discussion about the potential role transmission may play in some mitochondrial diseases. Recently, a sizable cohort of mitochondrial disease patients were assessed for neuromuscular transmission abnormalities in Braz et al. 2021 [3]. Overall, 78 patients with genetically confirmed mitochondrial disease were studied, and 20 displayed transmission defects by SFEMG (25.6%). This study found no correlation between abnormal transmission and neuropathy, but there was an increased risk of NMJ dysfunction with myopathy. However, previous work found an association with neuropathy and not myopathy [104]. Additionally, three patients without signs of myopathy or neuropathy displayed dysfunctional transmission—demonstrating that this defect can occur independently of both myopathy and neuropathy. NMJ dysfunction was not associated with severity nor was it limited to specific genotypes. While some genetic causes presented with higher rates of transmission dysfunction, no one cause saw 100% prevalence. The most prevalent mutations resulting in transmission disturbance were in RR2MB (three out of six patients), TWNK/C10orf2 (four out of nine patients), and the mtDNA mutation m.8344A>G (two out of five patients). Interestingly, both RR2MB and TWNK are implicated in mtDNA depletion syndrome wherein the copy number of mtDNA is severely depleted due to the impaired maintenance of the nucleotide pool and impaired mtDNA replication, respectively (reviewed in [105]). These were the only genes associated with mtDNA depletion syndrome, but their high levels of NMJ dysfunction point to the possibility that other genetic causes of this syndrome may suffer from transmission abnormalities as well. Additionally, both RR2MB and m.8344A>G mutations have been linked to impaired oxidative phosphorylation indicating that NMJ dysfunction may be due to compromised ATP synthesis [106,107,108,109]. However, many other mitochondrial diseases impair oxidative phosphorylation and ATP production and do not present with NMJ dysfunction, indicating that more research is needed in this area.

Previous studies have demonstrated wide-ranging frequencies of dysfunction, with ~27% to ~80% of observed patients demonstrating impaired transmission [104,110,111,112,113,114,115,116]. Some of these studies were small and/or performed within single families; however, they demonstrate how common transmission impairment is in mitochondrial disease. One limitation of the Braz et al. 2021 study is that all the included patients demonstrated signs of neuromuscular disease and may, therefore, be overestimating the prevalence of this dysfunction. However, one study found that 30% of mitochondrial patients without neuromuscular symptoms still present with transmission disturbance suggesting that this limitation may not be as impactful as suggested [104]. In a study of one familial cohort, with an unidentified genetic cause, 57% of the clinically unaffected participants displayed neuromuscular transmission impairment [104]. Thus, NMJ dysfunction is likely not limited to those with clinically apparent signs of neuromuscular symptoms; alternatively, neuromuscular fatigability may be underdiagnosed/underappreciated in mitochondrial disease.

While current mitochondrial genes linked to CMS are nuclear encoded, there is evidence that genes encoded in mtDNA are also involved in neuromuscular transmission. As briefly mentioned above, 2 out of 5 patients with an m.8344A>G mutation demonstrated impaired neuromuscular transmission—as did 3 out of 13 patients with large-scale mtDNA rearrangements and 2 out of 22 patients with m.3243A>G mutations [3]. Additionally, mtDNA deletions were found in 64% of patients diagnosed with myasthenia gravis and were most prevalent in serotypes without myasthenia gravis-associated antibodies [117]. The authors of this paper suggest that the true diagnosis of the patients without myasthenia gravis antibodies and with mtDNA deletions may be mitochondrial myopathy. Additionally, there are a few case reports of mtDNA deletions or mutations causing myasthenic symptoms [118,119]. This may indicate that mtDNA deletions or mutations alone can also cause myasthenia-like syndromes.

As previously mentioned, differential diagnosis can be challenging between mitochondrial myopathies and myasthenia gravis. SFEMG studies have shown that this is not only due to clinical presentation but the presence of increased neuromuscular jitter in both sets of patients. One study attempted to use SFEMG to distinguish myasthenia gravis and mitochondrial myopathy and found that, no matter how stringent the cut-off was, neuromuscular jitter was incapable of separating the two [113]. Other studies highlight the consequences of patients receiving the wrong diagnosis due to meeting SFEMG criteria for myasthenia gravis despite having mitochondrial myopathy [120,121,122]. For example, 12 patients were wrongly diagnosed with myasthenia gravis due to decremental EMGs, a positive response to injectable AChE inhibitors, or a partial response to oral AChE inhibitors [120]. In fact, the patients had mitochondrial myopathies, but this was only discovered after they had received thymectomies. Overlooking the connection between mitochondrial disease and the NMJ can have severe consequences for patients, and this should be kept in mind when diagnosing patients.

## 4. Discussion

These studies demonstrate a clear indication of the importance of the mitochondria at the NMJ, and that the NMJ is likely to be particularly sensitive to various dysfunctions of the mitochondria. This may be due to some of the roles of mitochondria highlighted above, including ATP synthesis and Ca^2+^ regulation, mitochondrial transport processes, or mtDNA transcription and replication. Understanding the role of mitochondria at the NMJ, as demonstrated in both clinical and biomedical research settings, is critical for proper patient care and continued research efforts. Due to the dependence of the NMJ on mitochondria, mutations that have milder effects may present first at the NMJ as it is particularly vulnerable. This may explain why patients with milder SLC25A1 and TEFM variants present with predominantly NMJ dysfunction. Due to this wide spectrum of disease presentations within mitochondrial gene mutations, it is difficult to determine whether a patient should be diagnosed with mitochondrial disease or a CMS. We propose that this distinction is made on a few criteria: (1) the primary symptoms for CMS patients should stem from NMJ dysfunction (demonstrated by tests such as SFEMG or RNS); (2) mitochondrial disease patients often demonstrate multi-system involvement. This line may be difficult to draw at times, and more discussion is likely needed among clinicians to establish standardized criteria.

The overlap in presentations, clinically and physiologically, between mitochondrial disease and CMS highlights the need to consider all possibilities when assessing patients. The clinical implications of not identifying a transmission defect were also demonstrated in a case study of a CMS patient misdiagnosed with mitochondrial myopathy [88]. Without treatment, the patient lost ambulatory abilities, which were regained following 2 months of treatment with salbutamol. Being aware of the differences, and the overlap, between mitochondrial disease and CMS is critical for proper patient care. Additionally, recognizing this overlap opens the door to mitochondrial disease patients with NMJ transmission defects trying drugs that have been effective in CMS. Admittedly, treatments typically aimed at CMS patients are not cure-alls for all forms of NMJ dysfunction [123]. However, the possibility of improvement should not be ignored, especially now that there are documented cases of mitochondrial CMS. Some patients with mitochondrial disease responded to AChE inhibitors, indicating they may be amenable to treatments targeting NMJ dysfunction [3,120,122]. While the response to AChE inhibitors is variable, so is the presence of transmission defects [115,121]. Salbutamol may also be an option to treat the complex NMJ defect caused by mitochondrial dysfunction as it resulted in a clinical improvement in patients with TEFM mutations [19]. We suggest using it as a first-line drug to explore in mitochondrial diseases with NMJ dysfunction. Available treatments for CMS are currently used off-label, but they are clinically approved. This provides a valuable group of drugs that mitochondrial patients may benefit from, particularly if they demonstrate signs of NMJ dysfunction.

While treatment options are currently limited for mitochondrial disease, should novel treatments be identified in the future mitochondrial CMS patients may also benefit. Additionally, as mitochondria contribute to many of the mechanisms impacted by other forms of CMS, targeting mitochondrial function in these other CMS types may be beneficial as well.

We propose that the extensive and diverse roles of mitochondria at the NMJ indicate that neuromuscular transmission defects from mitochondrial genes may be more common than previously thought. It is possible that the multi-systemic nature and, at times, the severe disease course of mitochondrial disorders have prevented the discovery of these transmission defects [18]. Additionally, as we know that deficiencies in mitochondrial functions can occur in non-mitochondrial disorder patients, there is the possibility of misdiagnosis [89].

### 4.1. SLC25A1 and TEFM CMS Demonstrate the Need for a Novel Subcategorization—Mitochondrial CMS

Finally, we encourage researchers and practitioners to consider a novel subcategory of CMS caused by mutations in mitochondria-associated genes. Mitochondria are involved in every aspect of the NMJ and attempting to specify one location for the pathomechanism with associated genes is unlikely to do anything other than narrow the scope prematurely. While the impact of mutations in SLC25A1 and TEFM on axon outgrowth seem to suggest a potential presynaptic pathomechanism, we believe it would be short-sighted to classify these two causative genes as solely presynaptic. The ubiquitous nature of mitochondria and the role of mitochondria in all aspects of the NMJ suggest that the function of these genes may be more multifaceted than currently understood. Additionally, functional studies of CHCHD10 demonstrate the potential of mitochondria-associated genes to cause NMJ dysfunction via a pre- and postsynaptic mechanism. However, the NMJ function has not yet been properly assessed in patients with pathogenic mutations in this gene and must be further investigated.

CMS caused by abnormal glycosylation has been added as a subcategory of CMS due to the inability to classify pathomechanisms based on location, and because patients have mostly presented with limb-girdle weakness and late-onset disease presentation [124]. In addition to fatigable muscle weakness and ptosis, both TEFM and SLC25A1 CMS-like patients have been reported to have intellectual disabilities and/or learning difficulties, which is uncommon in CMS patients [18,19]. Classifying mitochondrial CMS as a novel subtype will allow researchers, practitioners, and patients to identify commonalities in NMJ transmission defects that arise from impaired mitochondrial functioning. It will also raise awareness of the possibility of mitochondria contributing to neuromuscular transmission impairments, which may be important for clinical diagnostics, as well as treatment considerations for mitochondrial disease patients.

While this adds some complexity to the current categorization system, it allows researchers and clinicians to highlight a new pattern that is presenting itself in patients. Mitochondrial CMS patients may experience similarities in treatment response and presentation, both of which will be important clinically. To our knowledge, these two groups of patients have not all tried the same treatments. While 3,4-DAP had some reported success in an SLC25A1 patient and salbutamol had reported success in some TEFM patients, the use of each drug has not been reported in the other population [18,19]. It may be beneficial to compare the response to the same drugs to see if there is an overlap in response. These patients may also benefit from drugs targeting mitochondria, such as Coenzyme Q10, which restores the respiratory chain, or NAD+ boosters, which increase mitochondrial biogenesis and respiratory chain activity [125,126]. Overall, we believe that mitochondrial CMS deserves its own classification due to its uniqueness and the benefits it would provide.

### 4.2. Future Directions

Future work will need to focus on validating the pathomechanisms behind mitochondrial CMS and identifying mechanisms behind mitochondrial diseases with neuromuscular transmission defects. It would also be beneficial to provide an explanation as to why some cases of mitochondrial disease, caused by mutations to the same gene, result in NMJ involvement but not in others. The utilization of patient-derived induced pluripotent stem cell models or animal models expressing patient mutations may provide the opportunity to observe these differences in detail and assess the ability of the mitochondria within each sample to, for example, sequester Ca^2+^ or generate ATP. NMJ dysfunction should be considered as a tissue-specific presentation of mitochondrial diseases. Clinical NMJ involvement may be overlooked due to other severe symptoms; therefore, we suggest studying the NMJ with RNS and SFEMG as part of the diagnostic workup of patients with mitochondrial disease with fatigable muscle weakness and exercise intolerance. Furthermore, work should be conducted to investigate the translatability of treatment options across mitochondrial CMS types and in mitochondrial disease.

## 5. Conclusions

In conclusion, the literature demonstrates a clear and important role for mitochondria at the NMJ. The number of roles and their importance may represent a clinical vulnerability that is frequently overlooked. More moderate mutations in some genes may primarily affect the NMJ. The identification of two mitochondria-associated genes with CMS suggests a benefit of establishing a novel subcategory of CMS, mitochondrial CMS, to better define this group. The presence of NMJ defects in mitochondrial disease, and mitochondrial CMS, presents the opportunity to explore novel treatment options with patients to address these concerns. Finally, we encourage practitioners to keep mitochondrial CMS, as well as the possibility of NMJ transmission defects in mitochondrial disorders, in mind when dealing with patients, especially should they present with fatigable muscle weakness.

## Data Availability

Data sharing is not applicable for this article as no new data was generated.
